# RNA Coding and Transcriptional Regulation in Skin Repair: Insights from Single-Cell Profiling and Implications for Organoid-Based Regenerative Strategies

**DOI:** 10.3390/life16050784

**Published:** 2026-05-08

**Authors:** Edith Simona Ianoși, Daria Maria Tomoroga, Anamaria Todoran Butilă, Maria-Beatrice Ianoși, Anca-Meda Văsieșiu, Dorin Constantin Dorobanțu

**Affiliations:** 1 Department of Pulmonology, “George Emil Palade” University of Medicine, Pharmacy, Science and Technology of Târgu Mureș, 540139 Târgu-Mureş, Romania; edith.ianosi@umfst.ro; 2 “George Emil Palade” University of Medicine, Pharmacy, Science and Technology of Târgu Mureș, 540139 Târgu-Mureş, Romania; 3 Department of Genetics, “George Emil Palade” University of Medicine, Pharmacy, Science and Technology of Târgu Mureș, 540139 Târgu-Mureş, Romania; anamaria.todoran-butila@umfst.ro; 4 Clinic of Pulmonology, County Hospital Mures, 540011 Târgu Mures, Romania; ianosi.maria-beatrice@stud18.umfst.ro; 5 Department of Infectious Disease, “George Emil Palade” University of Medicine, Pharmacy, Science and Technology of Târgu Mureș, 540139 Târgu Mures, Romania; anca-meda.vasiesiu@umfst.ro; 6 Department of Plastic Surgery, “George Emil Palade” University of Medicine, Pharmacy, Science and Technology of Târgu Mureș, 540139 Târgu Mureș, Romania; dorin.dorobantu@umfst.ro

**Keywords:** skin regeneration, wound healing, single-cell transcriptomics, RNA regulation, skin organoids, fibrosis

## Abstract

Severe skin injury in humans typically heals through fibrotic remodelling rather than true regeneration, resulting in permanent loss of appendages, sensory function, and tissue architecture. Over the past decades, advances in bulk, single-cell, and spatial transcriptomic profiling have revealed that cutaneous wound repair is governed by dynamic, context-dependent gene-regulatory programmes across epidermal, dermal, vascular, and immune compartments. These studies highlight substantial heterogeneity in keratinocyte, fibroblast, and immune cell states, and identify RNA-mediated regulatory networks that bias healing toward either regenerative or fibrotic outcomes. In parallel, stem cell-derived skin organoids and advanced engineered skin equivalents have emerged as experimental platforms capable of reproducing key aspects of human skin organisation, offering new opportunities to move beyond purely reparative grafting strategies. This review integrates evidence from human or murine skin and wound transcriptomics, RNA-based regulatory mechanisms, and organoid-based skin engineering relevant to trauma and burn reconstruction. We summarise how protein-coding and non-coding RNAs (including miRNAs and lncRNAs) coordinate epithelial migration, inflammation resolution, angiogenesis, and ECM remodelling, and how the dysregulation of these networks contributes to pathological scarring. This article synthesises transcriptomic, RNA regulatory, and skin organoid research to propose a conceptual, hypothesis-generating framework for regenerative skin repair, without claiming clinical readiness or validated therapeutic translation.

## 1. Introduction

### 1.1. Scope and Background

Human skin is a complex, multilayered organ that provides an essential barrier against physical, chemical, microbial and ultraviolet factors, while also regulating thermoregulation, water homeostasis and immune surveillance [1]. The stratified epidermis, supported by a collagen- and elastin-rich dermis and a vascularised subcutis, maintains mechanical integrity and barrier function through tightly regulated programmes of keratinocyte proliferation, differentiation and desquamation, coupled to a dense network of resident immune cells, sensory nerves and adnexal structures [2].

Importantly, this review presents a conceptual and hypothesis-generating framework rather than a validated or ready-to-deploy therapeutic pipeline. Although the RNA-mediated regulation of wound healing and the generation of complex skin organoids are individually supported by experimental evidence, their integration into RNA-guided, patient-specific organoid-based therapies remains hypothetical and unproven in humans. Throughout this review, RNA expression profiles derived from bulk, single-cell, and spatial transcriptomic analyses are treated as descriptive readouts of cellular identity and state, whereas only RNA species with independent functional validation, such as specific miRNAs and lncRNAs, are discussed as causal regulatory levers capable of modulating wound-healing processes. By synthesising insights from single-cell and spatial transcriptomic profiling, RNA regulatory biology, and skin organoid engineering, this article aims to define evidence boundaries and translational design principles that may guide future experimental studies. The focus is on identifying regulatory principles and cellular states that could inform organoid design and shift skin repair from fibrotic closure towards regenerative healing, without implying direct clinical feasibility or causal translation to human skin regeneration. This review presents a conceptual and hypothesis-generating synthesis. It does not propose a clinically validated therapeutic strategy, nor does it imply that transcriptomic signatures can be directly translated into patient-specific graft design.

### 1.2. Clinical Burden of Burns and Traumatic Skin Loss

Major burns, extensive trauma and pressure injuries disrupt this finely tuned system, causing partial- or full-thickness loss of the integument and exposing the organism to fluid loss, infection, metabolic derangement and systemic inflammatory responses [3]. In acute severe burns or large traumatic defects, the combination of barrier failure, microbial contamination and intense inflammation can lead to sepsis, multi-organ dysfunction and death if immediate stabilisation of the wound environment is not achieved [4].

Following the acute phase, survivors of major burns and traumatic injuries often develop excessive fibrotic remodelling and pathological scarring that compromise long-term function [5,6].

Severe burns affect an estimated 11 million people annually, ranking among the leading causes of trauma-related morbidity worldwide, with 180,000 deaths reported each year, particularly in low-resource settings [7]. Patients with >40% TBSA burns face mortality rates exceeding 50%, even with early resuscitation and surgical intervention [8]. Trauma-related full-thickness skin loss is equally challenging: approximately 30–40% of major trauma admissions require reconstructive procedures that exceed the capacity of conventional autografts [9]. Survivors often experience long-term functional disability, psychological distress and need repeated surgeries, highlighting the urgent need for strategies that move beyond reparative closure toward regenerative features.

Furthermore, the economic burden of major burns is substantial, with prolonged hospitalisation, repetitive surgeries and rehabilitation contributing to some of the highest per-patient costs in acute care medicine, intensifying pressure on global healthcare systems [10].

### 1.3. Biological Barriers to Regeneration

Although split-thickness autografting and dermal substitutes remain the clinical gold standard for achieving rapid closure, these techniques do not fully replicate the native tissue complexity, as they lack vascular networks, innervation, hair follicles, and sweat glands, and therefore cannot produce a functional skin architecture [11,12]. Even when technically successful, grafted areas frequently evolve into hypertrophic scars or keloids, characterised by persistent myofibroblast activation, altered ECM remodelling and heightened mechanosensitivity, resulting in contractures, dysesthesia, restriction of movement and long-term disability [13,14].

A key biological limitation of adult human skin repair is the absence of appendage-restoring and scar-free regenerative programmes, with healing instead proceeding predominantly through fibrotic remodelling [15].

### 1.4. Limitations of Past Regenerative Approaches

Past regenerative approaches, including growth factor therapies, acellular dermal matrices, and cell-spray technologies, have also failed to restore full skin architecture because they cannot recreate coordinated multi-lineage development, vascularisation, and innervation, all of which are essential for functional skin repair [16,17].

These limitations underscore the need for strategies that move beyond isolated molecular or cellular interventions toward integrated graft design principles. In this context, this review synthesises convergent evidence to define testable design principles, hypothesising that wound-associated RNA signatures may inform the experimental preconditioning of organoid-derived skin constructs toward regenerative rather than fibrotic cellular states.

## 2. Materials and Methods

### 2.1. Search and Selection Criteria

The literature on skin organoids, engineered skin equivalents and advanced grafting strategies was identified using targeted searches in PubMed, Scopus and Web of Science with the keywords: “skin organoid”, “hiPSC skin”, “3D skin equivalent”, “full-thickness skin model”, “skin bioprinting”, “prevascularised graft”, and “transplantation of organoid-derived engineered skin constructs”. Conceptual and technical reviews were included when they proposed scalable frameworks or translational strategies relevant to trauma and burn reconstruction. Studies were excluded if they focused only on cosmetic or cancer modelling of skin, without regenerative or reconstructive relevance.

In addition to organoid studies, this review comprehensively examines transcriptomic and epigenomic datasets relevant to skin regeneration. Eligible studies used high-throughput molecular profiling methods, like bulk RNA-seq, scRNA-seq, spatial transcriptomics and/or small- or long-RNA-seq, applied to human skin, acute wounds, burns, chronic ulcers or translational animal models. Literature searches were conducted for peer-reviewed studies published between May 2005 and December 2025.

For non-coding RNA, inclusion required either genome-wide profiling with functional validation or well-defined mechanistic studies linking an lncRNA or miRNA to keratinocyte migration, differentiation, inflammation resolution or fibrosis in skin models. Validated examples include WAKMAR1 and HOXC13-AS in keratinocyte function.

This article is an integrative, evidence-based narrative review supported by a structured literature search and predefined inclusion criteria. It synthesises transcriptomic, regulatory RNA, and organoid-based studies relevant to skin regeneration, molecular regulation of skin repair, and translational reconstruction strategies, rather than performing a systematic review or meta-analysis.

### 2.2. Conceptual Definitions Relevant to Organoid-Based Skin Regeneration

Throughout the article, skin organoids are defined as stem cell-derived three-dimensional systems that self-organise through intrinsic developmental programmes to generate coordinated epidermal and dermal compartments, reflecting key aspects of native skin patterning rather than scaffold-imposed architecture [18,19]. Their defining property is emergent tissue organisation driven by developmental signalling cues.

The term organoid-based skin graft refers to a definitive, transplantable construct in which organoid-derived epithelial and mesenchymal populations actively contribute to long-term tissue replacement and integration, rather than serving as temporary wound coverage or passive biological dressings [20].

By contrast, an organoid-derived engineered skin construct refers to a transplantable, full-thickness skin equivalent. It is assembled using organoid-derived epithelial and/or mesenchymal cell populations within a structurally defined scaffold or matrix suitable for surgical handling and implantation. Importantly, most constructs proposed for clinical translation fall into the latter category rather than representing direct transplantation of intact skin organoids. In this review, RNA-guided refers to the experimental modulation or quality control assessment of a limited number of validated RNA regulators during in vitro organoid maturation.

### 2.3. Conceptual Framework for RNA-Guided Organoid-Based Skin Regeneration

#### 2.3.1. Conventional Versus Organoid-Based Approaches

Current reconstructive approaches were extensively compared with emerging organoid-based strategies regarding their ability to reproduce key attributes of native skin: appendage formation, vascular integration, neural ingrowth and potential for long-term functional regeneration. Table 1 summarises the evidence across graft types, using representative peer-reviewed studies to highlight both clinical strengths and inherent limitations. Autografts remain the clinical mainstay due to their reliability in achieving rapid closure, yet they consistently fail to restore adnexal structures, pigmentary units and sensory function, as confirmed by longitudinal morbidity analyses [21,22,23]. Allografts and xenografts serve only as temporary biological dressings, with predictable rejection and no contribution to permanent regeneration [24,25]. Dermal substitutes such as Integra^®^ Dermal Regeneration Template (Integra LifeSciences, Princeton, NJ, USA) [26,27] and Matriderm^®^ MatriDerm^®^ (MedSkin Solutions Dr. Suwelack AG, Billerbeck, Germany) [28,29] can improve surgical handling and reduce contracture formation, but they do not inherently regenerate adnexal structures or neural networks, and their integration is variable across defect size and anatomical region. Cultured epithelial autografts, intended to overcome donor-site limitations, also lack structural adnexal units and demonstrate inconsistent graft take and long-term fragility, especially in full-thickness defects [30,31].

Although these comparisons rely on robust clinical evidence for conventional grafts, the data supporting organoid-based approaches remain preclinical, and this discrepancy in evidence levels should be acknowledged when interpreting comparative regenerative potential.

In addition to classical grafting options, recent full-thickness engineered skin equivalents, such as prevascularised or bioprinted constructs, represent an intermediate category that improves early perfusion and structural stability but still fails to regenerate hair follicles, glands or neural networks.

In contrast, hiPSC-derived skin organoids demonstrate the first preclinical evidence of multi-lineage skin complexity, including the formation of epidermis, dermis, pigmented hair follicles, sebaceous glands and neural components within a single construct [32,33]. This suggests that the field is evolving beyond skin replacement toward constructs that exhibit regeneration-associated features.

These cumulative limitations across current reconstructive modalities underscore the need for grafts that can be molecularly programmed toward regenerative cell states, justifying the RNA-guided organoid strategies presented in the following section. Key characteristics, functional endpoints and translational readiness of conventional, engineered and organoid-based skin constructs are summarised in Table 1.

#### 2.3.2. Skin Organoids and Advanced In Vitro Models as Next-Generation Regenerative Tools

When pluripotent stem cells are exposed to stage-specific combinations of morphogens, they recapitulate developmental patterning cues and spontaneously segregate into lineage-restricted domains, giving rise to emergent structures that resemble embryonic organ primordia [47,48,49]. In the case of skin, hiPSC can be guided through ectodermal and neural crest fates using controlled modulation of BMP, Wnt and FGF signalling, leading to the co-development of stratified epidermis, dermal mesenchyme, melanocytes and hair follicle-like structures within a single three-dimensional aggregate [34,50].

At the same time, pluripotent stem cell-derived organoids and adult cell-based organotypic models have become increasingly sophisticated and are beginning to converge with organoid platforms in terms of complexity. Full-thickness human skin equivalents generated by seeding primary keratinocytes on top of fibroblast-populated collagen or fibrin hydrogels and culturing them at an air–liquid interface can now be prevascularised using ECs and pericytes, pre-innervated with sensory neurons and populated with immune cells such as Langerhans cells and T cells [40]. When such constructs are transplanted into preclinical models, pre-formed microvessels rapidly connect with host circulation, improving graft perfusion, survival and thickness compared with avascular sheets [41].

Crucially, skin organoids and advanced skin equivalents can provide capabilities that conventional split-thickness grafts inherently lack. Traditional autografts transfer only a thin portion of epidermis and superficial dermis, devoid of complete folliculo-sebaceous units, sweat glands and much of the native neural and vascular plexus, predisposing to dry, hairless, dysesthetic scars with limited thermoregulatory capacity. By contrast, organoid-derived skin constructs demonstrate the preclinical capacity to recapitulate features associated with regeneration, without yet achieving full functional restoration in humans [51,52].

Because organoids develop in a controlled environment, their composition can be tuned: the ratio of fibroblast subtypes, melanocyte density, and the presence or absence of specific immune or stromal components can, in principle, be adjusted before transplantation. This opens the possibility of generating grafts that are not only structurally complete but rationally engineered to minimise fibrosis, optimise integration and support long-term regenerative responses in patients with extensive burn or trauma-related skin loss [42,53].

Any organoid-based skin construct derived from hiPSCs or gene-modulated cells would likely be regulated as an ATMP under EMA and FDA guidelines, requiring GMP expansion, immunogenicity testing, genomic stability analysis, and long-term safety evaluation [54]. Ethical issues arise regarding donor consent, iPSC line ownership and equitable access, particularly in trauma and burn populations, where treatment windows are short [55,56]. These challenges must be addressed early to ensure translation is clinically viable, safe and scalable.

From a translational perspective, the initial clinical objective of organoid-based skin grafting is to achieve durable wound coverage with improved dermal organisation and reduced fibrotic remodelling, rather than the immediate restoration of fully mature adnexal structures. Over the longer term, the progressive maturation and functional integration of appendageal, vascular and neural components may further enhance tissue performance, supporting a shift from reparative closure toward features associated with regenerative healing.

#### 2.3.3. Toward RNA-Guided, Patient-Specific Organoid Grafts

Although current skin organoids already recapitulate many architectural and functional aspects of native skin, they are still largely generated using standardised protocols that do not fully account for inter-individual variation in cellular states, gene expression programmes and immune-inflammatory set points. However, high-throughput transcriptomic technologies have produced detailed atlases of human skin and wound tissue at single-cell resolution, revealing that keratinocytes, fibroblasts, ECs and immune populations exist on a spectrum of transcriptional states that differ between individuals, anatomic sites and disease contexts [19,20].

In human observational transcriptomic datasets, single-cell RNA-seq has identified distinct basal, suprabasal and appendage-associated keratinocyte subsets, multiple fibroblast lineages with spatially restricted signatures and resident immune cell populations poised for rapid activation [57]. Spatial transcriptomics has further mapped these transcriptional programmes back onto intact tissue architecture, demonstrating how gene expression gradients align with hair follicles, vasculature, adnexal units and mechanically stressed regions [58].

In the context of injury, single-cell and bulk RNA profiling have shown that acute burn and trauma wounds traverse highly stereotyped yet patient-modulated transcriptional trajectories, with early waves of inflammatory cytokines, chemokines and damage-associated molecular patterns followed by the induction of proliferative, angiogenic and matrix remodelling modules in regenerating keratinocytes and fibroblasts [59]. In non-regenerative outcomes such as hypertrophic scarring, some of these trajectories are distorted: fibroblasts adopt persistent myofibroblast states with elevated TGF-β-responsive gene expression, while keratinocytes may show aberrant differentiation and altered expression of junctional and barrier genes [60,61,62]. Importantly, these patterns are not uniform; inter-patient variability in gene expression modules, transcription factor activity and non-coding RNA networks is substantial, implying that a “one-size-fits-all” organoid graft will rarely be optimally matched to the host wound environment.

The emerging concept is that organoid design could be informed and customised by patient-specific RNA signatures. In research and delayed-reconstruction contexts, small biopsies obtained from wound margins or minimally affected donor skin may be subjected to single-cell RNA-seq and, where feasible, spatial transcriptomic analysis to characterise epidermal, dermal, and immune cell states associated with regenerative or fibrotic healing trajectories. Pluripotent stem cell-derived or adult skin-derived progenitors can be differentiated into skin organoids using standardised protocols that may be experimentally tuned, in research settings, based on regenerative or fibrotic transcriptional programmes identified from human wound transcriptomic datasets [63,64,65].

This RNA-centric approach does not imply the generation of fully individualised grafts at the level of every gene, but rather the experimental tuning of key regulatory modules and cell-state distributions toward regenerative states suggested by shared molecular features observed across patient groups. In contrast to conventional grafts, which are structurally constrained by donor site anatomy, organoid-derived skin constructs can be iteratively profiled and optimised during preclinical development, with single-cell transcriptomic analyses serving as research-grade quality control tools to verify regenerative-biased cellular states [66,67,68]. Ultimately, integrating transcriptomic insights from human wound datasets with organoid engineering offers a conceptual framework for developing skin grafts that are informed by regenerative molecular states rather than solely by surgical constraints.

Spatial transcriptomic analyses of human wound samples indicate that wound healing is organised into distinct molecular niches, with wound-edge regions enriched for activated keratinocytes, inflammatory signals and pro-fibrotic fibroblast states that are most relevant for graft integration. Complementary epigenomic analyses show that dermal fibroblasts can retain persistent chromatin states after injury, stabilising pro-scarring transcriptional programmes and contributing to recurrent fibrosis. Together, these findings suggest that organoid-derived grafts should be tuned to dominant wound-edge molecular states rather than reflecting averaged or homeostatic skin profiles.

#### 2.3.4. RNA Modulation as a Driver of Regenerative Healing

A growing body of evidence demonstrates that manipulating RNA programmes can causally shift adult skin from fibrotic toward regenerative healing. In murine and human wound models, blocking Engrailed-1 activation in fibroblasts—an adult pro-scarring lineage—induces a regenerative phenotype characterised by the restoration of dermal architecture, hair follicles and reduced scar formation [69]. En1 is a mechanosensitive transcription factor that marks pro-fibrotic fibroblasts during adult wound healing, and the inhibition of En1 activation shifts repair from scar formation toward regenerative healing with the restoration of normal skin architecture [70]. Similarly, miR-29 mimics, which repress collagen and ECM overproduction, significantly reduce dermal fibrosis and improve tissue organisation in preclinical models of skin injury [71].

Non-coding RNAs also exert direct control over epithelial repair: WAKMAR1, a keratinocyte-specific lncRNA essential for differentiation and migration, is markedly downregulated in chronic wounds, and its restoration rescues defective re-epithelialisation [72]. Anti-inflammatory miRNAs such as miR-146a further accelerate healing by tempering NF-κB-driven cytokine cascades and promoting angiogenesis [73,74]. Collectively, these findings support a causal regulatory role for specific RNA species under defined experimental conditions.

From a translational perspective, the feasibility of RNA-guided modulation in skin organoids depends not only on biological efficacy but also on delivery strategy, safety and quality control. Transient approaches, such as siRNA- or anti-sense oligonucleotide-based modulation, are generally more compatible with clinical-grade manufacturing than permanent genomic modification, as they enable the reversible tuning of cell states without altering genomic integrity. RNA delivery to three-dimensional skin constructs has been achieved using lipid-based nanoparticles, electroporation or scaffold-associated release systems, each presenting distinct trade-offs in efficiency, uniformity and scalability. Key translational risks include off-target gene regulation and innate immune activation through RNA-sensing pathways, underscoring the importance of careful sequence design and precise dose control. In this context, molecular quality control would be expected to include the verification of intended RNA expression changes, the assessment of downstream transcriptional effects and the confirmation of the absence of excessive inflammatory or stress-response signatures.

Taken together, these considerations position RNA-guided modulation as a tunable preconditioning strategy for organoid-derived skin constructs, while emphasising that delivery modality selection and molecular release criteria will be critical determinants of clinical feasibility. The stability of RNA-guided preconditioned states after transplantation remains uncertain. Because the wound bed is dynamic and exposes implanted constructs to hypoxia, inflammation, mechanical tension, microbial products and host-derived cytokines, in vitro-induced transcriptional states may be modified after implantation. Thus, RNA-guided preconditioning should be viewed as a strategy to bias early graft behaviour rather than permanently fix a regenerative phenotype, and its durability should be tested by longitudinal post-transplant single-cell and spatial transcriptomic profiling.

#### 2.3.5. Conceptual Framework for Staged Implantation of Organoid-Derived Engineered Skin Grafts

The reconstruction of extensive burns and traumatic full-thickness skin defects is traditionally guided by a staged paradigm in which wound stabilisation and definitive tissue replacement are temporally separated. Temporary biological coverage, including allo- or xenografts and bioengineered dermal templates such as Integra or MatriDerm, is used to restore barrier function, modulate the wound microenvironment and promote the formation of a vascularised neodermal bed before final reconstruction [24,75,76]. Importantly, these materials are not intended to remain permanently, but to condition the wound environment for a single, definitive grafting event.

This principle can be directly extended to organoid-based skin reconstruction. Rather than envisioning the repeated replacement or serial transplantation of organoids, organoid-derived grafts are conceptually best positioned as the terminal reconstructive step, analogous to definitive autografting [77,78]. Their role is not to serve as a temporary biological dressing, but to support durable engraftment and functional integration.

From an implantation perspective, organoid-based skin constructs could be applied either directly onto a well-prepared, vascularised wound bed or in combination with a dermal regeneration template. Dermal substitutes such as MatriDerm have been shown to provide mechanical stability, guide neodermis formation, and support vascular ingrowth, thereby creating a permissive interface for subsequent epithelial or composite grafting [79,80]. Within a regenerative framework, such matrices may serve as structural and biological supports beneath an organoid-derived epidermal–dermal construct, without competing with its long-term integration [46].

Importantly, the temporal requirements for producing organoid-informed skin constructs are compatible with established staged reconstruction protocols. Experimental studies demonstrate that stratified epidermal layers and dermal fibroblast-supported skin equivalents can be generated within approximately 4–6 weeks (for organoid-informed stratified epidermal–dermal equivalents) from human stem or progenitor cells, even though the emergence of complex appendages such as hair follicles requires longer culture periods. This timeframe overlaps with the clinically accepted duration of temporary wound coverage in severe burn care, during which biological dressings or dermal substitutes are routinely maintained before definitive grafting [81,82]. If a temporary allograft or xenograft is rejected and the patient’s clinical condition permits, temporary coverage may be re-established by the reapplication of an allo- or xenograft or a dermal substitute, thereby preserving wound stability and extending the window required for the generation of organoid-derived skin grafts before definitive implantation. These parallels do not imply clinical readiness or near-term implementation, but are discussed to illustrate conceptual compatibility with established reconstructive workflows.

In this framework, temporary biological grafts and dermal substitutes are used to stabilise and condition the wound bed, while the organoid-derived construct is reserved for definitive implantation once local and systemic conditions are favourable. Thus, the proposed strategy follows established staged reconstructive logic, while recognising that organoid-based definitive grafting remains preclinical and has not yet demonstrated comparative efficacy in humans. Accordingly, this framework is not intended to replace emergency wound closure in the acute burn phase. Its most realistic initial application is delayed or staged reconstruction after wound stabilisation, whereas acute use would likely require pre-manufactured, banked allogeneic or HLA-matched constructs rather than fully patient-specific autologous organoids.

#### 2.3.6. Transcriptional Modulation of Skin Organoids

Evidence from human organoid systems shows that cellular transcriptional states can be precisely engineered using protein and RNA-based tools. In skin organoids, modulating Wnt, BMP and FGF signalling alters lineage allocation and the emergence of appendage-forming compartments [83]. Human iPSC-derived skin organoids have been successfully patterned to produce fully pigmented hair follicles and complex dermal–epidermal architecture, demonstrating the plasticity of these systems to developmental cues [84]. Parallel studies in other organoid systems confirm that CRISPR/Cas9-based transcriptional activation or repression, as well as siRNA or anti-sense oligonucleotide delivery, can reliably reprogram cell states and gene-regulatory networks within 3D tissues [85,86]. These technologies are already used to modify epithelial differentiation, inflammatory responsiveness, and matrix interactions—mechanistic domains essential for wound repair. Together, these findings support the feasibility of molecularly programming organoids toward predefined regenerative RNA states before transplantation.

#### 2.3.7. Inter-Patient Transcriptomic Variability and Its Implications for Personalised Grafts

Transcriptomic analyses of human wounds demonstrate substantial inter-patient variability in cellular and molecular repair programmes. Human wound atlases demonstrate that individuals differ in their proportions of pro-fibrotic fibroblasts, inflammatory macrophage subtypes and basal keratinocyte progenitor pools, with these differences correlating strongly with divergent healing outcomes and scar severity [87,88]. Such inter-patient variability in wound-associated transcriptional programmes suggests that standardised grafts may not optimally interface with all host environments [89]. Instead, patient-specific RNA maps could guide targeted adjustments—such as reducing fibrotic fibroblast fractions or enhancing progenitor-rich keratinocyte states—to improve graft integration and reduce scarring. These datasets provide the mechanistic justification for personalising organoid composition based on individual transcriptomic profiles.

#### 2.3.8. Translational Positioning of Organoid-Derived Grafts

From a translational perspective, organoid-derived grafts should be regarded as definitive regenerative constructs intended for durable integration, rather than as fully mature replicas of adult skin at the time of implantation. Developmental and transplantation studies across organoid systems show that key aspects of functional maturation can continue in vivo after engraftment and do not require complete maturation in vitro before transplantation [90,91]. Accordingly, the clinical target product profile emphasises stable epidermal–dermal organisation, regenerative cellular states and surgical compatibility, rather than the immediate presence of fully developed appendages or adult-level tissue complexity [92].

Quality considerations, therefore, prioritise reproducible tissue architecture and appropriate lineage composition, rather than exhaustive functional maturity before implantation. Transcriptomic or phenotypic confirmation of regenerative-biased cellular states may serve as a pragmatic release principle, consistent with emerging frameworks for complex cell-based therapies [93]. Within the therapeutic landscape, organoid-derived grafts are positioned not as competitors to autografts or dermal substitutes, but as a complementary, terminal reconstructive option for extensive skin loss where current approaches achieve closure but not regeneration, offering an incremental and clinically compatible path toward improved long-term outcomes [94].

While the molecular, cellular, and engineering components discussed above are each supported by experimental evidence, their integration into a unified RNA-guided, patient-informed organoid-based skin regeneration pipeline remains hypothetical. In particular, the use of transcriptomic signatures to guide the preconditioning of organoid-derived engineered skin constructs, the extent to which such preconditioning can durably bias in vivo healing trajectories, and the scalability of these approaches within clinical timelines have not yet been demonstrated in humans. Accordingly, the proposed framework should be interpreted as a conceptual synthesis informed by the existing transcriptomic and organoid literature.

## 3. Evidence Synthesis

### 3.1. Single-Cell and Spatial RNA Profiling

Bulk and single-cell RNA-seq studies have demonstrated that cutaneous wound healing proceeds through tightly regulated transcriptional trajectories spanning inflammatory, proliferative and remodelling phases, and that the dysregulation of these programmes is associated with impaired or fibrotic healing outcomes [95]. Single-cell analyses of murine wounds revealed that fibroblasts adopt diverse lineage-restricted transcriptional states, including pro-regenerative and pro-fibrotic populations, with TGF-β-responsive myofibroblast states tightly linked to scar formation [96].

In murine lineage-tracing models, a landmark single-cell study demonstrated that specific fibroblast lineages drive fibrotic versus regenerative repair, showing that the blockade of Engrailed-1-associated programmes prevents scarring and promotes re-generation [97]. Together, these findings support the concept that fibroblast heteroge-neity and dynamic state transitions critically influence wound outcome.

Large-scale spatial and single-cell genomic mapping of human skin has further revealed that keratinocytes, immune cells, ECs and fibroblasts inhabit discrete tran-scriptional niches that vary across anatomical location and age, underscoring that wound repair is shaped by microenvironment-specific gene expression programmes rather than uniform tissue-wide states [98]. These findings provide a cellular map for organoid engineering by identifying wound-associated epithelial, stromal, vascular, and immune states that may need to be enriched, avoided, or experimentally modu-lated in future constructs.

### 3.2. RNA-Based Regulation of Cutaneous Wound Repair

Several lncRNAs and miRNAs with validated roles in keratinocyte migration, angiogenesis, inflammation, and fibrotic remodelling—including WAKMAR1, HOXC13-AS, CASC2, lnc-URIDS, miR-21, miR-29 family members, and miR-146a—have been identified in wound-healing models and are summarised in Table 2 [72,99,100,101,102,103,104].

Together, these RNA regulators illustrate how post-transcriptional control can bias wound-healing trajectories toward regenerative or fibrotic outcomes. In this context, validated RNA regulators may serve not only as mechanistic markers of wound repair, but also as candidate modulatory targets for organoid preconditioning.

### 3.3. Implications for Organoid Design

Single-cell studies show that wound healing depends on dynamic regenerative or fibrotic cell states, particularly within fibroblast lineages, which strongly influence whether skin regenerates or scars [105,106]. These findings support the rationale for incorporating regenerative-biased cellular states into organoid-derived skin constructs. RNA regulators validated in wound healing, including the lncRNA WAKMAR1, which promotes keratinocyte migration, and miRNAs such as miR-21, miR-29, and miR-146a, which respectively modulate proliferation, fibrosis, and inflammation [107,108], provide molecular tools to precondition organoids toward regenerative behaviour. Integrating RNA-guided cues during organoid maturation may bias graft cellular states toward reduced fibrosis and improved compatibility with the wound environment.

### 3.4. Experimental Validation of an RNA-Guided Organoid-Based Regeneration Conceptual Framework

The feasibility of an RNA-guided, organoid-based regenerative strategy is supported by experimental evidence that independently validates each major step of the proposed conceptual framework. Human skin transcriptomic atlases have defined molecular niches relevant to wound repair and fibrosis [109]. At the same time, lncRNA-focused profiling and mechanistic studies in human skin models have demonstrated that specific lncRNAs act as regulators of re-epithelialisation by modulating keratinocyte migration, proliferation and differentiation. The dysregulation of these lncRNA-controlled programmes is associated with delayed wound closure and prolonged inflammatory signalling in acute and chronic wounds [110,111]. Complementary work on miRNAs has established that miRNAs form coordinated networks influencing keratinocyte behaviour, inflammation, angiogenesis and matrix remodelling in cutaneous wounds and chronic ulcers, providing an additional layer of RNA-based control points [112,113].

On the engineering side, hPSCs have been guided through the defined modulation of BMP, FGF and TGF-β signalling to self-organise into complex skin organoids containing stratified epidermis, dermis, hair follicles, sebaceous glands, adipocytes and sensory neurones, which can generate planar, hair-bearing human skin after xenotransplantation into immunodeficient mice [35]. Building on this, 3D bioprinting has been used to assemble “skin organoid” constructs from human keratinocytes, fibroblasts and ECs that are geometrically matched to full-thickness defects and, when applied to immunodeficient mice, significantly accelerate wound closure, enhance epithelialisation and vascularisation and dampen excessive inflammatory responses compared with controls [38]. Most compellingly, the transplantation of hiPSC-derived engineered skin in a murine frostbite model not only accelerated closure but also reduced early inflammation, increased epidermal stem cell fractions and limited fibroblast-to-myofibroblast transition and fibrotic remodelling, demonstrating that organoids can actively reshape wound-healing trajectories in vivo rather than merely survive as passive grafts [36]. Recent reviews synthesise these advances in organoid biology and engineering, highlighting how skin organoids have evolved from simple layered epidermis to cyst-like appendage-bearing constructs and how 3D printing and microfluidics can be combined with omics-level characterisation to standardise and upscale such systems [37,114].

**Figure 1:**
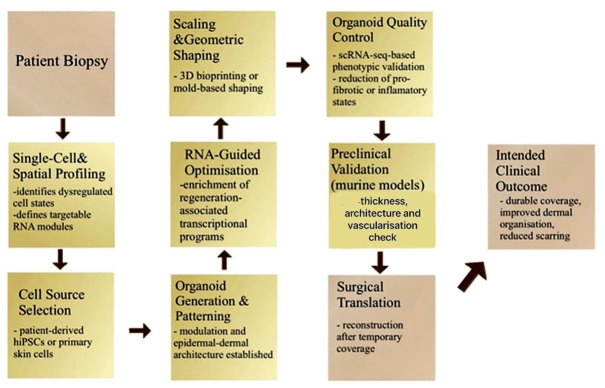
Conceptual synthesis of independently validated components. Pale beige panels indicate laboratory and preclinical steps (molecular profiling, organoid generation, etc.), while light golden yellow panels denote surgical phases (biopsy, reconstruction and intended clinical outcomes). The figure synthesises experimentally supported components rather than depicting a validated clinical protocol. Arrows indicate the sequential progression of the proposed workflow.

Together, these studies show that patient-level molecular profiling, organoid generation and scaling, in vivo functional benefit and RNA-based control of key wound-healing programmes are all experimentally supported, so that the remaining challenges for an RNA-guided organoid therapy are now largely integrative, translational [43] and regulatory, rather than biological. Key facts are shown in the image below. Figure 1 summarises the experimentally supported steps of an RNA-guided, organoid-based skin regeneration conceptual framework, integrating transcriptomic profiling, organoid engineering, RNA modulation and preclinical validation.

## 4. Discussion

### 4.1. RNA Coding and Regulatory Networks in Skin Regeneration

Regenerative versus fibrotic skin healing is governed by coordinated RNA regulatory networks that operate across transcriptional and post-transcriptional layers, rather than by isolated gene expression changes [115]. Protein-coding RNAs define core cellular functions such as keratinocyte differentiation, ECM synthesis, and angiogenic signalling, while non-coding RNAs modulate the timing, magnitude, and resolution of these responses through diverse regulatory mechanisms [116]. miRNAs act as buffering regulators that stabilise gene expression programmes and constrain excessive inflammation or matrix deposition, thereby limiting pathological scarring [117]. Similarly, lncRNAs function as molecular scaffolds, decoys, or transcriptional regulators that shape cell-state transitions during tissue repair and regeneration [118].

Importantly, RNA-mediated regulation is highly dynamic and phase-specific during wound healing, coordinating inflammatory activation, epithelial migration, and stromal remodelling across distinct temporal windows [119]. The dysregulation of these RNA networks can lock cells into persistent inflammatory or pro-fibrotic states, contributing to defective repair and excessive scar formation [120]. In the context of organoid-based skin engineering, RNA-guided modulation offers a reversible and tunable strategy to align cellular transcriptional states with regenerative programmes without permanent genomic alteration. This positions RNA regulatory networks as actionable control points for preconditioning skin organoids toward regenerative phenotypes, supporting their conceptual suitability for personalised regenerative graft design.

### 4.2. Preclinical Evidence Supporting Organoid-Based Skin Regeneration

A widely cited study by Lee et al. [34] demonstrated that complex human skin-like tissue, including stratified epidermal and dermal compartments and appendage-associated structures, can be generated in vitro from pluripotent stem cells. Upon transplantation into immunodeficient mice, these constructs generated planar, hair-bearing skin-like tissue that integrated with host vasculature and persisted long-term, providing strong preclinical evidence of functional integration in vivo.

Together, these findings demonstrate the feasibility of complex skin-like tissue formation in preclinical models. However, human evidence currently remains strongest for transcriptomic mapping and in vitro skin modelling. In contrast, functional proof of organoid-derived graft integration, vascularisation and regenerative benefit remains largely limited to murine xenograft or injury models. Subsequent studies extended these findings by applying organoid-based or organoid-informed constructs to experimental wound repair models, as summarised in Table 3.

Evidence supporting the RNA-mediated regulation of skin regeneration derives from human transcriptomic analyses, in vitro human skin models and predominantly murine in vivo studies. While murine models have been central to defining fibroblast heterogeneity and fibrotic versus regenerative programmes, species-specific differences in skin biology mean that lineage markers such as Engrailed-1 do not have direct equivalents in humans. Accordingly, the translational framework proposed here emphasises conserved regulatory modules and cell states identified in human wounds, rather than the direct transfer of animal lineage definitions, for programming human-derived organoid systems before transplantation.

### 4.3. Immunology and Immunogenicity Considerations in Organoid-Derived Grafts

Organoid-derived skin grafts introduce complex multicellular constructs into an immunologically active tissue, making host-graft immune interactions a central translational consideration. Skin is densely populated by resident immune cells, including Langerhans cells, dermal dendritic cells and tissue-resident T cells, which rapidly sense cellular stress and non-self signals following transplantation [121,122]. Even autologous or HLA-matched cell products may elicit inflammatory responses due to culture-induced stress, altered differentiation states or damage-associated molecular patterns released during implantation [120].

Preclinical studies across organoid and engineered tissue platforms indicate that graft survival and integration depend not only on antigenicity but also on early inflammatory control and timely vascularisation, which together limit hypoxia-driven immune activation [123]. Accordingly, immunogenicity in this context should be viewed as a continuum of immune engagement, rather than binary rejection, reinforcing the need to evaluate local inflammation, immune cell infiltration and cytokine signatures alongside classical histocompatibility considerations [124].

### 4.4. Surgical Interface and Host Integration Considerations

From a clinical perspective, the success of organoid-based skin grafts may depend as much on surgical interface conditions as on intrinsic graft biology. Extensive burn and traumatic wounds present hostile microenvironments characterised by inflammation, hypoxia, bacterial burden and altered perfusion, all of which can impair early graft survival if not adequately controlled at the time of implantation [125]. Preclinical studies of complex skin substitutes consistently demonstrate that rapid vascular inosculation and stable physical contact with a well-prepared wound bed are critical determinants of engraftment and long-term tissue integrity [39,125].

Current experimental strategies to improve the vascularisation of engineered and organoid-derived skin constructs focus mainly on creating a pre-existing vascular compartment before implantation, rather than relying exclusively on slow host-driven angiogenesis. Tremblay et al. demonstrated that endothelialised reconstructed skin containing keratinocytes, fibroblasts and endothelial cells formed capillary-like structures in vitro and, after transplantation into nude mice, these structures contained mouse blood within less than 4 days. In contrast, non-endothelialised reconstructed skin required approximately 14 days to achieve comparable vascularisation, supporting early inosculation as a key mechanism of graft perfusion [41]. Another strategy is the use of decellularised vascular scaffolds combined with bioreactor perfusion: Groeber et al. generated a vascularised human skin equivalent by seeding human dermal microvascular endothelial cells, fibroblasts and keratinocytes onto a biological vascular scaffold, producing endothelial cell-lined, perfusable vascular structures together with a stratified epidermal compartment [126]. More recently, 3D bioprinting has enabled the spatial organisation of vascular and stromal components within multilayered skin constructs; Baltazar et al. printed human keratinocytes, fibroblasts, endothelial cells and pericytes, showing the in vitro self-assembly of interconnected microvascular networks and the in vivo inosculation of human endothelial cell-lined structures with host mouse microvessels after implantation [127]. Together, these primary experimental studies indicate that prevascularisation, perfusable vascular scaffolds and bioprinted endothelial–pericyte networks are the most relevant current approaches to accelerate vascular integration, although all remain preclinical and require further validation before translation to human burn reconstruction.

Unlike temporary biological dressings, definitive regenerative grafts must tolerate early mechanical stress, fluid shear and immune surveillance while establishing vascular and stromal continuity with host tissue. Experimental evidence from engineered skin and composite graft models indicates that excessive motion, poor bed vascularity or persistent inflammation at the graft–host interface predispose to partial graft loss, delayed integration and fibrotic remodelling [128,129]. These observations support the view that organoid-based skin constructs should be implanted only after adequate wound conditioning, infection control and perfusion optimisation have been achieved, consistent with established reconstructive principles.

### 4.5. Limitations and Potential Obstacles to Clinical Translation

#### 4.5.1. Biological Limitations

The clinical translation of hiPSC-derived skin constructs also requires careful consideration of safety risks inherent to pluripotent cell-based products. Key concerns include the presence of residual undifferentiated cells, genomic instability acquired during expansion, and long-term tumorigenicity following transplantation. Accordingly, established safety frameworks for pluripotent-derived ATMP emphasise stringent lineage purification, genomic integrity assessment, and extended in vivo engraftment studies before clinical use. These requirements are consistent with existing regulatory expectations and represent critical quality thresholds rather than unique barriers to organoid-derived grafts.

In parallel, clinically relevant biological failure modes must be considered, including susceptibility to infection and biofilm formation during prolonged wound preparation [130], delayed or insufficient vascular inosculation that may compromise graft survival [44], and dysregulated fibroblast activation that can drive fibrotic remodelling rather than regeneration if cellular composition and transcriptional states are not adequately controlled [131,132].

#### 4.5.2. Engineering and Manufacturing Limitations

Although each component of an RNA-guided, organoid-based regenerative strategy has been demonstrated independently, several barriers remain before it can be fully translated into clinical practice. A central limitation is the absence of an integrated and standardised workflow combining patient-specific transcriptomic profiling, organoid generation, RNA-based modulation and in vivo validation into a single, streamlined conceptual framework, a challenge shared across organoid-based therapeutic platforms [44,130].

Standardisation, therefore, remains a major priority. Although single-cell RNA-seq offers a powerful framework for molecular quality control, consensus transcriptional benchmarks defining a “regenerative” skin organoid have not yet been established, and cross-laboratory reproducibility remains limited [133,134]. Practical considerations related to production time, cost and logistical complexity [particularly in acute injury settings] also pose significant challenges [44].

#### 4.5.3. Clinical and Translational Limitations

Clinical translation is also constrained by the time required to generate organoid-derived skin constructs relative to wound-care timelines. While staged reconstruction in major burns may enable a window of several weeks before definitive grafting, fully autologous iPSC-based approaches remain challenging in acute settings, making banked HLA-matched lines or applications in elective reconstruction and chronic wounds more realistic initial pathways.

The assessment of organoid-derived skin grafts requires clearly defined outcome measures beyond wound closure. Relevant endpoints include standardised scar scores, markers of fibrosis, barrier function and long-term structural integration to distinguish transient repair from durable functional improvement.

Nonetheless, advances in hiPSC biobanking, accelerated differentiation protocols, scalable manufacturing strategies and evolving regulatory frameworks for ATMP suggest that many of these constraints are addressable [128]. Taken together, these considerations indicate a field that is still maturing rather than fundamentally constrained, with core biological principles established and the remaining obstacles largely integrative, technical and regulatory in nature.

## 5. Conclusions

Human skin repair following severe injury is dominated by fibrotic remodelling rather than true regeneration, reflecting fundamental constraints in adult wound healing. Advances in bulk, single-cell, and spatial transcriptomic profiling have revealed that repair outcomes are governed by heterogeneous, dynamic, and spatially organised gene-regulatory programmes across epidermal, dermal, vascular, and immune compartments. These studies indicate that regenerative and fibrotic healing are associated with distinct cellular states and RNA regulatory networks rather than uniform repair trajectories.

At the same time, stem cell-derived skin organoids and advanced engineered skin equivalents have emerged as experimental platforms capable of recapitulating key aspects of native skin architecture. Although these systems remain preclinical, they provide a controllable context in which cellular composition and transcriptional states can be systematically interrogated and manipulated.

This review synthesises current evidence to outline a conceptual framework in which transcriptomic and RNA regulatory insights define testable design principles for future organoid-derived skin constructs. Importantly, this framework does not represent a validated therapeutic strategy. Substantial challenges remain in translating descriptive transcriptomic signatures into actionable regulatory targets and in demonstrating durable functional benefit in vivo. Nonetheless, by integrating human transcriptomic data with organoid biology, this work provides a structured foundation for future experimental efforts aimed at shifting skin repair from fibrotic closure toward regenerative healing.

## Figures and Tables

**Table 1:** Evidence map of representative organoid-based and engineered skin constructs. Supporting references for each construct type are cited in the main text. Light blue was used for preclinical organoid-based or engineered skin constructs, medium blue for in vitro constructs and dark blue for clinical constructs.

Construct Type	Cell Source	Key Features	Model	Primary Endpoints Assessed	Major Limitations	Evidence Level/Translational Readiness
hiPSC-derived skin organoids [32,33,34,35]	Human-induced pluripotent stem cells	Stratified epidermis and dermis, pigmented hair follicle-like structures, sebaceous gland-like units, rudimentary neural components	Mouse xenograft	Long-term engraftment, hair-bearing skin formation, vascular inosculation	Limited scalability, long culture time, immunogenicity considerations, immature appendages at implantation	Preclinical (proof-of-concept)
Organoid-derived planar skin constructs [34,35,36,37]	Human-induced pluripotent stem cells	Epidermal–dermal architecture with appendage-forming potential	Mouse xenograft	Barrier formation, tissue integration, hair cycling	Limited defect size, manufacturing complexity, GMP scalability not established	Preclinical
Bioprinted organoid-informed skin constructs [37,38,39]	Human keratinocytes, fibroblasts and endothelial cells	Full-thickness geometry, spatially organized epidermal and dermal compartments, pre-organized vascular structures	Mouse full-thickness wound	Accelerated wound closure, enhanced epithelialization, improved vascularisation	Absence of appendage regeneration, limited immune integration	Preclinical/early translational
Engineered full-thickness skin equivalents (prevascularized) [40,41,42,43,44]	Primary human keratinocytes, fibroblasts and endothelial cells	Pre-formed microvascular networks, dermal structural support	Mouse graft	Improved perfusion, graft thickness and survival	Lack of hair follicles, sweat glands and neural networks	Advanced preclinical
Skin-on-chip and microfluidic skin models [37,45]	Primary human skin cells	Barrier function, perfusion control, immune and inflammatory modelling	In vitro	Barrier integrity, permeability, vascular perfusion	Not transplantable; research and modelling applications only	In vitro (research tool)
Cultured epithelial autografts (CEA) [17,30,31,46]	Primary human keratinocytes	Epidermal coverage	Human clinical use	Wound closure, graft survival	Fragility, lack of dermis and appendages, high scarring risk	Clinical (coverage only)

**Table 2:** Representative lncRNAs and miRNAs implicated in cutaneous wound repair. For each RNA, the primary cellular context, functional role, and experimental model system in which activity has been validated are indicated. Evidence derives from a combination of human in vitro or ex vivo models and preclinical murine studies, as cited.

RNA	RNA Type	Primary Cell Type(s)	Functional Role in Skin Repair	Model System(s)	Key Refs
WAKMAR1	lncRNA	Keratinocytes	Promotes keratinocyte migration and differentiation; supports re-epithelialization	Human ex vivo skin models	[98]
HOXC13-AS	lncRNA	Keratinocytes	Regulates keratinocyte differentiation-associated transcriptional programs	Human in vitro models	[99]
CASC2	lncRNA	Endothelial cells	Promotes angiogenesis and granulation tissue formation via miR-155/HIF-1α axis	Diabetic wound models (murine)	[100]
lnc-URIDS	lncRNA	Dermal fibroblasts	Inhibits collagen maturation through PLOD1, delaying wound repair	Diabetic wound models (murine)	[101]
miR-21	microRNA	Fibroblasts, keratinocytes	Promotes keratinocyte migration and granulation; excessive activity contributes to fibrosis	Murine wound models	[102]
miR-29 (family)	microRNA	Dermal fibroblasts	Represses collagen and ECM gene expression; anti-fibrotic	Murine models; human incisional trial	[103]
miR-146a	microRNA	Immune cells (macrophages), endothelial cells	Suppresses NF-κB signaling; limits inflammation and promotes angiogenesis	Murine diabetic and chronic wound models	[104]

**Table 3:** Representative preclinical and review-based studies supporting the feasibility of organoid-based skin constructs. All experimental evidence summarised is derived from in vivo murine models or in vitro systems. Review articles are included to contextualise technological maturity and translational considerations rather than to provide primary efficacy data. Light blue indicates primary experimental studies, whereas medium blue indicates review articles.

Source	Evidence Type	Main Contribution	Relevance to This Review
Zhang et al., 2024 [38]	Primary experimental study	3D-bioprinted human-derived skin organoids accelerated repair of full-thickness skin defects in immunodeficient mice.	Supports preclinical feasibility of bioprinted skin organoid constructs for wound repair.
Wang et al., 2025 [36]	Primary experimental study	hiPSC-derived skin organoids combined with gelatin hydrogel promoted repair in a mouse frostbite injury model.	Supports preclinical evidence that skin organoids can modulate injury repair responses.
Hong et al., 2023 [33]	Review article	Summarised development, construction methods, applications, prospects, and limitations of bioengineered skin organoids.	Provides background and technological context.
Zeng et al., 2025 [37]	Review article	Reviewed engineered skin organoid models, including development, construction strategies, applications, and challenges.	Provides conceptual and translational context.

## Data Availability

No new data were created or analysed in this study.

## Abbreviations

ATMP	Advanced Therapy Medicinal Product
BMP	Bone morphogenetic protein
CASC2	Cancer susceptibility candidate 2 [lncRNA]
ECM	Extracellular matrix
EMA	European Medicines Agency
FGF	Fibroblast growth factor
GMP	Good Manufacturing Practice
HLA	Human leukocyte antigen
hPSCs	Human pluripotent stem cells
hiPSC	Human induced pluripotent stem cell
iPSC	Induced pluripotent stem cell
lncRNA	Long non-coding RNA
miRNA	MicroRNA
NF-κB	Nuclear factor kappa-light-chain-enhancer of activated B cells
RNA-seq	RNA sequencing
scRNA-seq	Single-cell RNA sequencing
siRNA	Small interfering RNA
TBSA	Total body surface area
TGF-β	Transforming growth factor beta
WAKMAR1	Wound and keratinocyte migration-associated lncRNA 1
Wnt	Wingless/Integrated signalling pathway
EC	Endothelial cell
EMT	Epithelial–mesenchymal transition
lnc-URIDS	Long non-coding RNA associated with ulcer-related inflammatory dermal signalling
